# Levels of Lysozyme and SLPI in Bronchoalveolar Lavage: Exploring Their Role in Interstitial Lung Disease

**DOI:** 10.3390/ijms25084297

**Published:** 2024-04-12

**Authors:** Rubén Osuna-Gómez, Maria Mulet, Silvia Barril, Elisabet Cantó, Paloma Millan-Billi, Ana Pardessus, David de la Rosa-Carrillo, Diego Castillo, Silvia Vidal

**Affiliations:** 1 Inflammatory Diseases, Institut de Recerca Sant Pau (IR Sant Pau), 08041 Barcelona, Spain; rosuna@santpau.cat (R.O.-G.); mmulet@santpau.cat (M.M.); ecanto@santpau.cat (E.C.); 2 Respiratory Department, Institut de Recerca Biomèdica de Lleida (IRBLleida), Hospital Universitari Arnau de Vilanova-Santa María, Translational Research in Respiratory Medicine, Universitat de Lleida (UdL), 25198 Lleida, Spain; silvia.barril@gmail.com; 3 Department of Respiratory, Hospital de la Santa Creu i Sant Pau, 08041 Barcelona, Spain; pmillanbilli@gmail.com (P.M.-B.); apardessus@santpau.cat (A.P.); drosa@santpau.cat (D.d.l.R.-C.); dcastillo@santpau.cat (D.C.); 4 Department of Respiratory, Hospital Universitario Germans Trias i Pujol, 08916 Barcelona, Spain

**Keywords:** fibrosis, AMPs, immune cells, lysozyme, SLPI

## Abstract

Interstitial lung diseases (ILDs) are characterized by inflammation or fibrosis of the pulmonary parenchyma. Despite the involvement of immune cells and soluble mediators in pulmonary fibrosis, the influence of antimicrobial peptides (AMPs) remains underexplored. These effector molecules display a range of activities, which include immunomodulation and wound repair. Here, we investigate the role of AMPs in the development of fibrosis in ILD. We compare the concentration of different AMPs and different cytokines in 46 fibrotic (F-ILD) and 17 non-fibrotic (NF-ILD) patients by ELISA and using peripheral blood mononuclear cells from in vitro stimulation in the presence of lysozyme or secretory leukocyte protease inhibitor (SLPI) from 10 healthy donors. We observed that bronchoalveolar lavage (BAL) levels of AMPs were decreased in F-ILD patients (lysozyme: *p* < 0.001; SLPI: *p* < 0.001; LL-37: *p* < 0.001; lactoferrin: *p* = 0.47) and were negatively correlated with levels of TGF-β (lysozyme: *p* = 0.02; SLPI: *p* < 0.001) and IL-17 (lysozyme: *p* < 0.001; SLPI: *p* < 0.001). We observed that lysozyme increased the percentage of CD86^+^ macrophages (*p* < 0.001) and the production of TNF-α (*p* < 0.001). We showed that lysozyme and SLPI were associated with clinical parameters (lysozyme: *p* < 0.001; SLPI: *p* < 0.001) and disease progression (lysozyme: *p* < 0.001; SLPI: *p* = 0.01). These results suggest that AMPs may play an important role in the anti-fibrotic response, regulating the effect of pro-fibrotic cytokines. In addition, levels of lysozyme in BAL may be a potential biomarker to predict the progression in F-ILD patients.

## 1. Introduction

Interstitial lung disease (ILD) comprises a variety of diffuse lung alterations characterized by different patterns of inflammation and fibrosis [1,2]. This idiopathic pulmonary fibrosis (IPF) is often accompanied by significant morbidity and mortality, with a median survival of 5 years after diagnosis [3]. During fibrosis, epithelium-directed injury due to inflammation, infection, or exposure to airway pollutants [4] may lead to the recruitment of immune cells and the induction of new tissue formation through transforming growth factor beta (TGF-β), IL-10, and IL-17 [5,6]. TGF-β can be produced during tissue damage repair in the lungs by alveolar macrophages, bronchial epithelium, and hyperplastic type II alveolar epithelial cells (AECs) [7]. The mechanism behind is the activation of the TGF-β pathway. This is due to a loss of regenerative capacity in damaged epithelial cells, converging to epithelial–mesenchymal transition and enhancing the pro-fibrotic process [8,9,10]. IL-10 can be synthesized during tissue-damage repair by B cells, macrophages, dendritic cells, and multiple subsets of T cells [6]. As well, IL-17A can be secreted during inflammation and tissue-damage repair by various cell types, including Th17 lymphocytes and epithelial cells [11]. Furthermore, one outcome of epithelium-directed injury is the production of antimicrobial peptides (AMPs) by epithelial cells, macrophages, and neutrophils [12].

When pathogen invasion occurs, AMPs are triggered, enabling them to directly eliminate invading microorganisms and indirectly kill them by activating the immune system [13,14]. These peptides are constitutive or induced effector mediators with antimicrobial activity through multiple mechanisms, including protein degradation (lysozyme), nutrient depletion (lactoferrin), cellular disruption and lysis, and the inhibition of virulence factors (LL-37, SLPI) [15]. Further, AMPs have a dual role in immune activation [16,17]. First, AMPs can activate innate immune cells, including neutrophils and macrophages, leading to the production of cytokines or chemokines [18]. This activation enhances the ability of these immune cells to engulf and kill pathogenic microorganisms [19]. Secondly, AMPs have the capacity to activate dendritic cells to present antigens to T cells, consequently inducing the activation of cytotoxic T cells [19] to eliminate pathogens [19,20,21,22].

Some studies have performed an analysis of AMPs in BAL in chronic pulmonary diseases [21,22]. Findings reported that lysozyme, lactoferrin, secretory leukocyte proteinase inhibitor (SLPI), and LL-37 are prominent and abundant AMPs within the airway, exhibiting altered expression patterns in chronic lung diseases [19,20,21,22]. It has been reported that BAL from IPF patients had lower levels of SLPI and β-defensin when compared to that in control subjects [23]. Moreover, other studies showed that neutrophilic inflammation was associated with SLPI deficiency or inactivation in IPF patients, which impaired wound repair, altered the microbiome, and resulted in a higher disease burden [22]. In addition, TGF-β levels in the lungs of IPF patients may have contributed to these lower levels of SLPI [24]. Overall, these studies indicate that lower concentrations of specific AMPs are linked to a poorer prognosis in ILD patients.

With the objective of determining the role of AMPs in ILD patients, we firstly compared the bronchoalveolar lavage (BAL) levels of AMPs in fibrotic (F-ILD) and non-fibrotic (NF-ILD) patients. We then studied the association between the levels of these AMPs and the leucocyte subpopulations present in BAL and the concentration of pro- and anti-inflammatory local cytokines. In addition, we analyzed the functional mechanisms of these AMPs using in vitro assays and cultures with recombinant lysozyme and SLPI. Concretely, we cultured macrophages and CD4^+^ T cells with AMPs to determine changes in the production of TGF-β, IL-10, and IL-17. Finally, we studied the association between AMP levels and clinical parameters and disease progression.

## 2. Results

### 2.1. Population

Sixty-three patients were included in this study. A summary of patient characteristics is given in Table 1. Mean age was 67 years and there was a predominance of male gender (41 cases). A total of 46 cases (73%) were classified as F-ILD. Regarding diagnosis, the most frequent diagnoses were connective tissue disease-associated ILD (CTD-ILD), followed by IPF and smoking-related ILD (SR-ILD).

### 2.2. BAL Lysozyme, SLPI, and LL-37 Were Decreased in F-ILD Patients and Associated with Clinical Features

**Figure 1:**
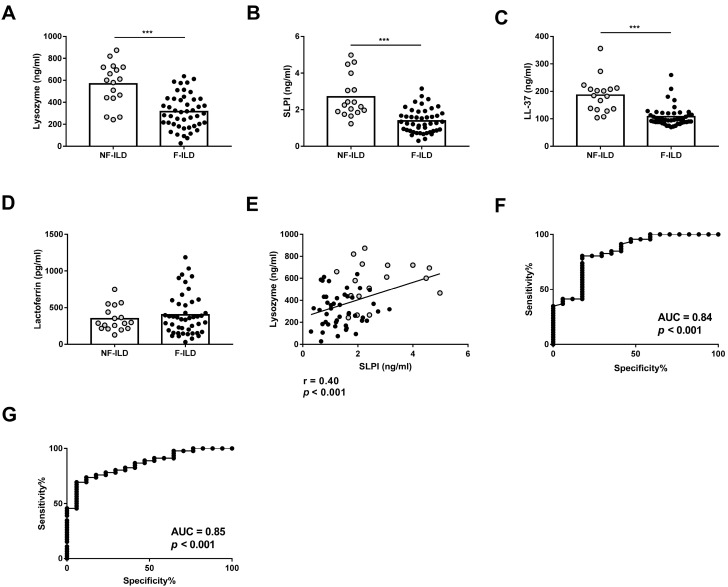
BAL levels of (**A**) lysozyme, (**B**) SLPI, (**C**) LL-37, and (**D**) lactoferrin determined by ELISA in fibrotic (F-ILD) and non-fibrotic (NF-ILD) patients. (**E**) Correlation between levels of lysozyme and SLPI in BAL from ILD patients. (**F**) ROC curve analysis of lysozyme levels to distinguish F from NF patients. (**G**) ROC curve analysis of SLPI levels to distinguish F from NF patients. Spots in black correspond to F-ILD patients and spots in gray to NF-ILD patients. *** *p* < 0.001.

We compared the concentration of AMPs in the BAL of F-ILD and NF-ILD patients by ELISA. Lysozyme, SLPI, and LL-37 levels were significantly higher in NF-ILD than in F-ILD patients (lysozyme: 567 ± 46.7 vs. 316 ± 23.0 pg/mL, *p* < 0.001; SLPI: 2.71 ± 0.27 vs. 1.38 ± 0.09 ng/mL, *p* < 0.001; LL-37: 186 ± 15.3 vs. 107 ± 5.32 ng/mL, *p* < 0.001, respectively) (Figure 1A–C). In contrast, lactoferrin levels were comparable in both groups (Figure 1D). We also found a positive correlation between lysozyme and SLPI levels (r = 0.40, *p* < 0.001), but no correlation between other AMPs (Figure 1E).

To find a statistically valid cut-off point of lysozyme levels to differentiate F-ILD from NF-ILD patients, an ROC curve analysis with all patients was performed, leading to <262.7 pg/mL as the value for F-ILD patients. The area under the ROC curve was 0.84, sensitivity was 41.3%, and specificity was 94.12% (Figure 1F). The valid cut-off off point of SLPI levels to differentiate F-ILD from NF-ILD patients showed <1.638 as the value for F-ILD patients. The area under the ROC curve was 0.85, sensitivity was 69.5%, and specificity was 94.12% (Figure 1G).

**Figure 2:**
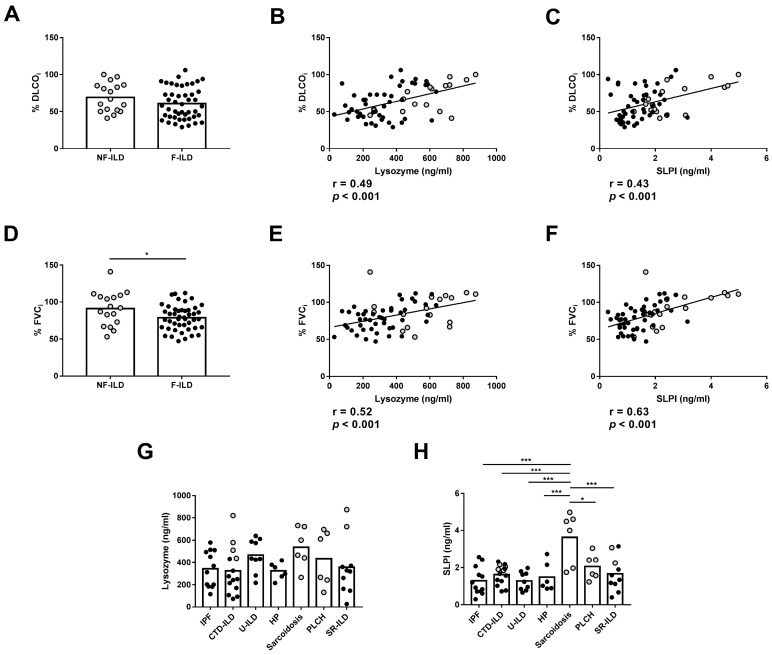
Comparison of lysozyme and SLPI levels in baseline features of ILD patients. (**A**) Comparison of initial percentage of DLCO (%DLCOi) between fibrotic (F-ILD) and non-fibrotic (NF-ILD) patients. Correlation between %DLCOi and (**B**) lysozyme and (**C**) SLPI levels in BAL from ILD patients. (**D**) Comparison of initial percentage of FVC (%FVCi) between F-ILD and NF-ILD patients. Correlation between %FVCi and (**E**) lysozyme and (**F**) SLPI levels in BAL from ILD patients. Comparison of (**G**) lysozyme and (**H**) SLPI levels by etiology (IPF: idiopathic pulmonary fibrosis; CTD-ILD: connective tissue disease-associated interstitial lung disease; U-ILD: unclassificable interstitial lung disease; HP: hypersensitivity pneumonitis; PLCH: pulmonary langerhans cell histiocytosis; SR-ILD: smoking-related interstitial lung disease). Spots in black correspond to F-ILD patients and spots in gray to NF-ILD patients. * *p* < 0.05; *** *p* < 0.001.

We then analyzed the association between the AMPs and the clinical features of the patients at baseline. Furthermore, no differences were observed in %DLCOi when compared between F-ILD and NF-ILD patients (Figure 2A). We found a positive correlation between initial percentage of diffusing capacity for carbon monoxide (%DLCOi) and SLPI (r = 0.43, *p* < 0.001) or lysozyme (r = 0.49, *p* < 0.001) levels in all patients (Figure 2B,C). Furthermore, when analyzing the F and NF groups separately, we demonstrated a positive correlation between %DLCOi and SLPI levels (r = 0.30 *p* = 0.04 and r = 0.53 *p* = 0.02, respectively) or lysozyme levels (r = 0.33 *p* = 0.02 and r = 0.60 *p* = 0.01, respectively). We also found that the initial percentage of forced vital capacity (%FVCi) was decreased more in F-ILD than NF-ILD patients (78.8 ± 2.50 vs. 91.2 ± 5.54, *p* = 0.04, respectively) (Figure 2D). There was a positive correlation between %FVCi and SLPI (r = 0.63, *p* < 0.001) and lysozyme (r = 0.52, *p* < 0.001) levels in all patients (Figure 2E,F). Furthermore, when analyzing the F and NF groups separately, we observed a positive correlation between %FVCi and SLPI levels (r = 0.46, *p* = 0.001 and r = 0.58, *p* = 0.01, respectively). However, lysozyme levels showed a positive correlation only in F patients (r = 0.45, *p* = 0.001), while in the NF group, the correlation was not significant.

When we classified patients according to disease etiology, higher SLPI levels were found in Sarcoidosis than in other etiologies (Figure 2G), and levels of lysozyme were similar in all groups (Figure 2H).

### 2.3. Association of Lysozyme and SLPI with Pro- and Anti-Inflammatory Cytokines in the BAL of ILD Patients

**Figure 3:**
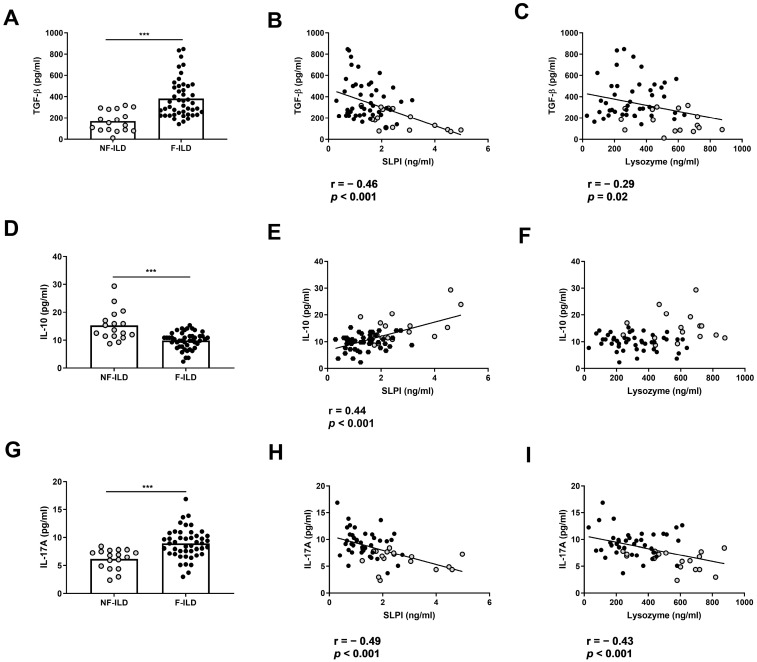
Association of lysozyme and SLPI levels with pro- and anti-inflammatory cytokines in ILD patients. (**A**) Comparison of TGF-β levels in BAL of fibrotic (F-ILD) and non-fibrotic (NF-ILD) patients. Correlation between TGF-β and (**B**) SLPI and (**C**) lysozyme levels in BAL from ILD patients. (**D**) Comparison of IL-10 levels in BAL of F-ILD and NF-ILD patients. Correlation between IL-10 and (**E**) SLPI and (**F**) lysozyme levels in BAL from ILD patients. (**G**) Comparison of IL-17 levels in BAL of F-ILD and NF-ILD patients. Correlation between IL-17 and (**H**) SLPI and (**I**) lysozyme levels in BAL from ILD patients. Spots in black correspond to F-ILD patients and spots in gray to NF-ILD patients. *** *p* < 0.001.

To analyze whether AMP levels were associated with a pro- or anti-inflammatory profile in BAL from ILD patients, we determined the BAL concentration of TGF-β, IL-10, and IL-17. The levels of TGF-β in BAL were increased in F-ILD when compared to that in NF-ILD patients (382 ± 26.6 vs. 170 ± 23.7 pg/mL, *p* < 0.001, respectively) (Figure 3A) and were negatively correlated with SLPI (r = −0.46, *p* < 0.001) or lysozyme (r = −0.29, *p* = 0.02) levels in the BAL of ILD patients (Figure 3B,C). In contrast, the levels of IL-10 in BAL were decreased in F-ILD when compared to those in NF-ILD patients (9.80 ± 0.43 vs. 15.2 ± 1.30 pg/mL, *p* < 0.001, respectively) (Figure 3D) and correlated with SLPI (r = 0.44, *p* < 0.001) (Figure 3E) but not with lysozyme levels in the BAL of ILD patients (Figure 3F). The levels of IL-17 in BAL were increased in F-ILD when compared to NF-ILD patients (8.93 ± 0.39 vs. 6.18 ± 0.43 pg/mL, *p* < 0.001, respectively) (Figure 3G) and were negatively correlated with SLPI (r = −0.49, *p* < 0.001) and lysozyme (r = −0.43, *p* < 0.001) levels in the BAL of ILD patients (Figure 3H,I).

**Figure 4:**
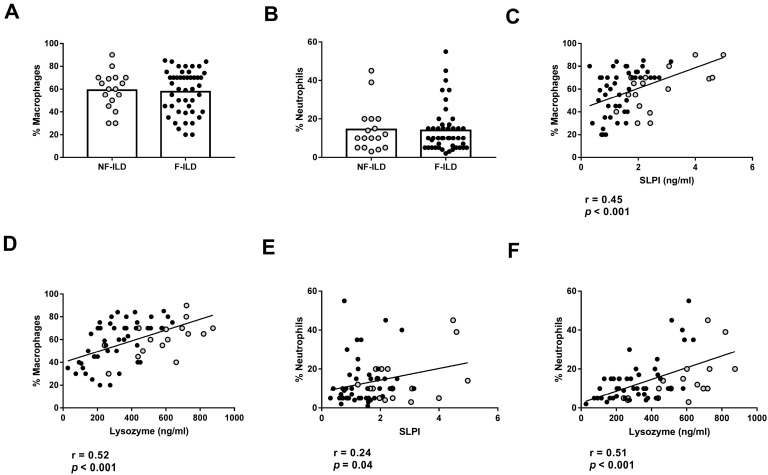
Association of lysozyme and SLPI levels with leucocyte populations in BAL from ILD patients. (**A**) Comparison of percentage of macrophages in BAL of fibrotic (F-ILD) and non-fibrotic (NF-ILD) patients. Correlation between percentage of macrophages and (**B**) SLPI and (**C**) lysozyme levels in BAL from ILD patients. (**D**) Comparison of percentage of macrophages in BAL of F-ILD and NF-ILD patients. Correlation between percentage of neutrophils and (**E**) SLPI and (**F**) lysozyme levels in BAL from ILD patients. Spots in black correspond to F-ILD patients and spots in gray to NF-ILD patients.

We then analyzed the association between AMP levels and the different immune cell subpopulations in the BAL of ILD patients. The percentages of macrophages and neutrophils in BAL were similar between F-ILD and NF-ILD patients (Figure 4A,B) and correlated with SLPI (%macrophages: r = 0.45, *p* < 0.001; %neutrophils: r = 0.24, *p* = 0.04) and lysozyme (%macrophages: r = 0.52, *p* < 0.001; %neutrophils: r = 0.51, *p* < 0.001) levels in the BAL of ILD patients (Figure 4C,F). Furthermore, when analyzing the F and NF groups separately, we demonstrated a positive correlation between the percentage of macrophages and SLPI (r = 0.50 *p* < 0.001 and r = 0.51 *p* = 0.03, respectively) or lysozyme levels (r = 0.60 *p* < 0.001 and r = 0.66 *p* = 0.004, respectively). Moreover, when analyzing the F and NF groups separately, we observed a positive correlation between the percentage of neutrophils and lysozyme levels (r = 0.48, *p* = 0.001 and r = 0.71, *p* < 0.001, respectively). However, SLPI levels did not show a significant correlation in F and NF patients.

### 2.4. Lysozyme and SLPI Increased CD86^+^ Macrophages and Decreased IL-17 Production In Vitro

**Figure 5:**
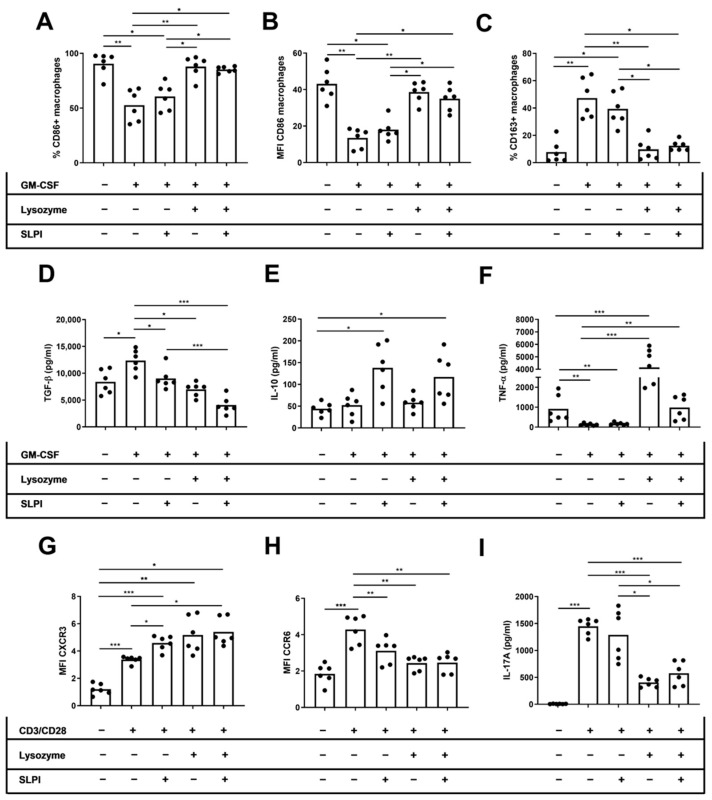
Effect of lysozyme and SLPI on phenotype and cytokine secretion by activated macrophages and T cells. For macrophage experiments, HD PBMCs were cultured for seven days in the presence of M-CSF in the presence of lysozyme, SLPI, or both cytokines. For T cell experiments, HD PBMCs were cultured for 72 h in the presence of CD3/CD28 activator in the presence of lysozyme, SLPI, or both cytokines. (**A**) Percentage of CD86^+^ macrophages. (**B**) MFI of CD86 on macrophages. (**C**) Percentage of CD163^+^ macrophages. (**D**) Levels of TNF-α on macrophage culture supernatant. (**E**) Levels of TGF-β on macrophage culture supernatant. (**F**) Levels of IL-10 on macrophage culture supernatant. (**G**) MFI of CXCR3 expression on CD4^+^ T cells. (**H**) MFI of CCR6 expression on CD4^+^ T cells. (**I**) Levels of IL-17A on T cell culture supernatant. * *p* < 0.05; ** *p* < 0.01; *** *p* < 0.001.

We performed in vitro studies to directly examine the effect of lysozyme and SLPI on cytokine production by innate and adaptive immune cells. We cultured M2 macrophages or activated T cells derived from HD with recombinant SLPI, lysozyme, or a combination of both molecules. Afterwards, we conducted an approximate assessment of the M1 and M2 macrophages, as well as the Th1 and Th17 T cells. On M2 macrophages, we found that only lysozyme increased the percentage and MFI of CD86 on macrophages (%CD86: 52.6 ± 5.90 vs. 87.8 ± 3.99, *p* = 0.003; MFI CD86: 13.4 ± 2.19 vs. 34.9 ± 2.72, *p* = 0.02, respectively), independent of the SLPI effects (%CD86: 52.6 ± 5.90 vs. 85.3 ± 1.11, *p* = 0.01; MFI CD86: 13.4 ± 2.19 vs. 38.6 ± 2.29, *p* = 0.007, respectively) (Figure 5A,B). Moreover, the percentage of CD163^+^ macrophages only decreased when cells were cultured in the presence of lysozyme (47.3 ± 5.90 vs. 9.75 ± 3.26, *p* = 0.003), independently of the SLPI effects (47.3 ± 5.90 vs. 12.4 ± 1.53, *p* = 0.01) (Figure 5C). TGF-β levels in M2 macrophage culture supernatant decreased when cells were cultured in the presence of lysozyme, SLPI, or both cytokines (13,354 ± 851 vs. 6999 ± 529, *p* = 0.01; 13,354 ± 851 vs. 9035 ± 816, *p* = 0.04; 13,354 ± 851 vs. 4068 ± 643 pg/mL, *p* < 0.001, respectively) (Figure 5D). However, IL-10 levels in the culture supernatant did not change when cells were cultured in the presence of SLPI, lysozyme, or both cytokines (Figure 5E). TNF-α levels in the culture supernatant increased when cells were cultured in the presence of lysozyme or both cytokines (100 ± 23.1 vs. 4145 ± 694, *p* < 0.001; 100 ± 23.1 vs. 983 ± 143 pg/mL, *p* = 0.004, respectively) (Figure 5F). On activated CD4^+^ T cells, the MFI of CXCR3 increased but the MFI of CCR6 decreased when cells were cultured in the presence of lysozyme, SLPI, or both cytokines (MFI CXCR3: 3.37 ± 0.10 vs. 5.17 ± 0.54, *p* = 0.03; 3.37 ± 0.10 vs. 4.58 ± 0.22, *p* = 0.04; 3.37 ± 0.10 vs. 5.39 ± 0.40, *p* = 0.03, respectively) (MFI CCR6: 4.28 ± 0.32 vs. 2.43 ± 0.15, *p* = 0.003; 4.28 ± 0.32 vs. 3.11 ± 0.28, *p* = 0.008; 4.28 ± 0.32 vs. 2.46 ± 0.21, *p* = 0.003, respectively) (Figure 5G,H). The levels of IL-17A in activated CD4^+^ T cell culture supernatant decreased when cells were cultured in the presence of lysozyme, independent of the SLPI effects (1448 ± 61.5 vs. 405 ± 34.8, *p* < 0.001; 1448 ± 61.5 vs. 575 ± 90.9 pg/mL, *p* < 0.001, respectively) (Figure 5I).

### 2.5. Lysozyme and SLPI Levels Were Associated with Clinical Disease Progression

**Figure 6:**
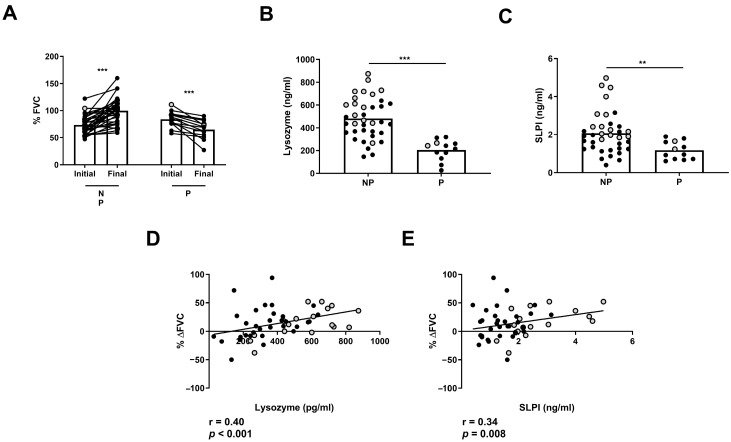
Differences in FVC progression of ILD patients according to lysozyme and SLPI levels. Comparison of (**A**) initial and final FVC percentage in progressors (P) and non-progressors (NP) in ILD patients. Comparison of (**B**) lysozyme and (**C**) SLPI according to FVC in P and NP ILD patients. Correlation between percentage of FVC progression (% ΔFVC) and levels of (**D**) lysozyme and (**E**) SLPI in BAL from ILD patients. Spots in black correspond to F-ILD patients and spots in gray to NF-ILD patients. ** *p* < 0.01; *** *p* < 0.001.

Next, we analyzed whether AMP levels can indicate clinical disease progression in ILD patients. First, we classified non-treated ILD patients in two groups: those with a stable or improved FVC (NP) progression and those with a significant decrease in FVC progression (P) between the initial and final measurement after 1 year of follow-up (Figure 6A). We found that NP patients had increased levels of BAL lysozyme and SLPI compared to P patients (482 ± 29.7 vs. 204 ± 25.9 pg/mL, *p* < 0.001; 2.07 ± 0.17 vs. 1.17 ± 0.13 ng/mL, *p* = 0.01, respectively) (Figure 6B,C). Furthermore, we found a correlation between %ΔFVC and BAL levels of lysozyme (r = 0.40, *p* < 0.001) or SLPI (r = 0.34, *p* = 0.008) in ILD patients (Figure 6D,E).

## 3. Discussion

In the current study, we found significant differences in AMP concentrations in the BAL from F-ILD and NF-ILD patients. In addition, BAL levels of lysozyme and SLPI levels were associated with clinical parameters and with lower levels of cytokines involved in the fibrotic process. We were able to establish a functional relationship between AMPs and cytokines when we found that lysozyme and SLPI reduced the in vitro production of TGF-β and IL-17. Globally, our results suggest a role for AMPs in anti-fibrotic response, regulating the production and hence the effect of crucial cytokines.

Here, we found that lysozyme, SLPI, and LL-37 levels in BAL were lower in F-ILD than in NF-ILD patients. This finding is in line with other studies that showed lower SLPI levels in IPF patients than HD in BAL [23]. Despite that, there are no studies examining BAL levels of LL-37 and lysozyme in ILD patients; LL-37 and lysozyme have been reported to have an anti-fibrotic effect in vitro, reducing proliferation, extracellular matrix, and collagen genes in human fibroblasts [13,25,26]. One explanation for our findings is that the activation of a damaged epithelium in F-ILD patients, due to chronic inflammation, induced a decreased production of these AMPs, impeding complete wound repair. Another explanation may be that the exacerbated proteolytic activity of dysregulated enzymes, a finding of chronic lung diseases, reduced AMPs [27]. Our results are consistent with the described anti-fibrotic role of lysozyme, SLPI, and LL-37 in other murine models of fibrotic diseases [12,28].

We showed that higher levels of lysozyme and SLPI were positively associated with improved DLCO and FVC in ILD patients. To our knowledge, there are no studies that associate BAL levels of lysozyme or SLPI with these clinical parameters in ILD patients. However, our findings are in line with previous reports of a positive correlation between another AMP, such as dual oxidase 1 (DUOX1), and better spirometry and DLCO in chronic obstructive pulmonary disease (COPD) patients [29]. Moreover, lysozyme administration improved FVC by suppressing inflammation in the small airways of COPD patients [30]. We can speculate that one mechanism of respiratory function improvement by AMPs is by stimulating the host defense to protect from epithelial injury due to distorting lung microbiome. Otherwise, a persistent distorting lung microbiome would lead to a continuous epithelial injury and to the alteration of pulmonary function. This is consistent with studies that indicate that certain lung microbiomes correlate with alveolar inflammation and clinical outcomes in pulmonary fibrosis [27]. It has been reported that IPF patients exhibited less phylogenetic alpha diversity in their lower airway microbiome and were associated with changes in the epithelial barrier and the levels of antimicrobial peptides [23].

Our findings suggest that lysozyme and SLPI have an immunomodulatory effect. First, we found a negative correlation between IL-17 and SLPI and lysozyme levels in BAL, which have been reinforced by our in vitro results. In line with this, other authors have shown that lysozyme significantly reduced the expression of IL-17 in dextran sodium sulfate-induced colitis [31]. Furthermore, SLPI plays a role in inhibiting NF-kB binding, which leads to a decreased production of proinflammatory cytokines, including IL-17 [32].

Second, we found a negative correlation between TGF-β and SLPI and lysozyme levels in BAL, again reinforced by our in vitro results. SLPI has been shown to decrease TGF-β levels by regulating macrophage activation [33,34]. In regard to lysozyme, our in vitro results suggest that this AMP induces the differentiation of macrophages to a M1 phenotype, increasing TNF-α and decreasing TGF-β production. Another study has also shown that lysozyme decreased TGF-β production by retinal pigment epithelial cells through a reduction of T regulatory (Treg) cell differentiation [35].

Third, we showed that lysozyme and SLPI levels were higher in patients with less pulmonary progression. In this line, lysozyme administration improved FVC by suppressing inflammation in the airways of COPD patients [30]. In addition, it has been reported that SLPI inhibits airway hyperresponsiveness through IL-17A regulation [36]. A possible mechanistic explanation of our findings is that AMPs induce pro-healing effects on injured tissue by suppressing mediators [37].

Despite the contribution of these results to the understanding of the role of AMPs in ILD, our study has some limitations. One is that our in vitro findings cannot be fully extrapolated to BAL cells. Additionally, to give consistency to our in vitro macrophage results, it would be interesting to perform a deeper phenotyping of macrophage subtypes. Another limitation is that we did not analyze the levels of AMPs in the plasma of these patients. It would be interesting to compare the levels of AMPs in plasma and BAL collected at the same time point to understand the connection between these two compartments. Finally, more types and larger cohorts with ILD patients should be included to validate lysozyme and SLPI levels as prognostic biomarkers.

## 4. Materials and Methods

### 4.1. Patients and Design

This is a prospective observational study that was conducted at Hospital de la Santa Creu i Sant Pau, Barcelona (Spain), from 2014 to 2019. The population of this study consisted of new patients referred to an ILD clinic, in whom a bronchoalveolar lavage was performed as part of their diagnostic assessment. Patients whose diseases originated from various etiologies [38] were presented at the ILD multidisciplinary meeting to confirm ILD diagnosis based on current guidelines [39,40,41]. Patients were managed according to the current recommendations by their physicians, regardless of their participation in this study. For each patient, written informed consent was obtained, and ethical approval for this study was granted by the Hospital de la Santa Creu i Sant Pau Institutional Ethics Committee.

Cases were classified as fibrotic ILD (F-ILD) or non-fibrotic ILD (NF-ILD) [42] based on visual analysis of the findings of a chest high-resolution computed tomography (HRCT). Those cases with a predominance of fibrotic features (reticulation, traction bronchiectasis, and honeycombing) were grouped as F-ILD. The following variables were collected for each patient: age, sex, smoking habit, diagnosis, and treatment started after multidisciplinary assessment. Lung function test (LFT) values, including diffusing capacity for carbon monoxide (DLCO) and forced vital capacity (FVC), were collected from baseline and at the last follow-up appointment as vital status. In addition, the results of the main BAL cell subpopulations (by flow cytometry analysis) were collected.

### 4.2. Processing of BAL

Bronchoscopy with BAL was performed for diagnostic purposes according to the European Respiratory Society BAL Task Force Group guidelines [43]. Briefly, BAL was filtered through sterile gauze and centrifuged at 2000 rpm for 10 min at room temperature. The supernatant was then stored at −20 °C.

For the phenotyping of BAL samples, cells were stained with anti-CD3-FITC (Immunotools Friesoythe, Germany), anti-CD4-Viogreen (Miltenyi Biotec, Bergisch Gladbach, Germany), anti-CD56-PE (Immunotools), anti-CD14-PE-Vio615 (Miltenyi Biotec), anti-CD45-PE-Cy7 (BD Biosciences, San Jose, USA), anti-CD8-APC (Immunotools), and anti-CD20-APC-Cy7 (Miltenyi Biotec). CD45^+^ cells were gated to select leukocytes. We identified CD4^+^ T cells (CD3^+^ CD4^+^ CD8^−^), CD8^+^ T cells (CD3^+^ CD4^−^CD8^+^), NK cells (CD3^-^CD56^+^), and B cells (CD3^−^CD56^−^CD20^+^) on lymphocytes, gating leukocytes by size and complexity. Macrophages were identified by size, complexity, and CD14^+^ expression, and neutrophils were identified by size, complexity, and CD14^−^ as previously described in our laboratory [44]. Data acquisition and analyses were performed on a MACSQuant Analyzer 10 flow cytometer (Miltenyi Biotec) using FlowJo version 10 (FlowJo, Ashland, OR, USA).

### 4.3. Cell Culture and Phenotyping by Flow Cytometry

Peripheral blood from 6 healthy donors (HDs) was collected in Vacutainer tubes with heparin (BD Biosciences). Peripheral blood mononuclear cells (PBMCs) were isolated from heparinized peripheral blood using a Ficoll-Histopaque gradient (Lymphoprep, AXIS-SHIELD PoCAs, Oslo, Norway).

For T cell culture, freshly isolated PBMCs were cultured in 96-well plates (Thermo Fisher Scientific, Vienna, Austria) with RMPI-1640 (Biowest, Nuaille, France) supplemented with 10% FBS and 1% Penicillin/Streptomycin (Biowest) in the presence of dynabeads human T-activator CD3/CD28 (Thermo Fisher Scientific, Hillsboro, OR, USA) with 4 µg/mL SLPI (R&D Systems, Minneapolis, MN, USA), 5 ng/mL of lysozyme (Sigma Aldrich, St. Louis, MO, USA), or a combination of both cytokines for 72 h in 5% CO_2_ at 37 °C. For macrophage culture, freshly isolated PBMCs were cultured in 96-well plates with complete RMPI-1640. After two hours, non-adherent cells were removed by repeated washing and the remaining adherent fraction was cultured in the presence of 10 ng/mL of macrophage colony stimulating factor (M-CSF; Immunotools) with 4 µg/mL SLPI (R&D Systems, Minneapolis, MN, USA), 5 ng/mL of lysozyme (Sigma Aldrich, St. Louis, MO, USA), or a combination of both cytokines for 7 days in 5% CO_2_ at 37 °C. Prior to analysis, cell culture supernatant was collected and stored and measured by ELISA for cytokine production determination.

For T cell staining, cells were analyzed by flow cytometry using anti-CD4-Viogreen (Miltenyi Biotec), anti-CD8-PE (Immunotools), anti-CD14-FITC (Immunotools), anti-CXCR3-PE-Vio770 (Miltenyi Biotec), and anti-CCR6-APC (Miltenyi Biotec) antibodies. For macrophage staining, cells were analyzed by flow cytometry using anti-CD86-FITC (Immunotools), anti-CD163-PE (BD Biosciences), and anti-CD14-APC (Immunotools) antibodies.

Data acquisition and analysis were performed on a MACSQuant Analyzer 10 flow cytometer (Miltenyi Biotec) using FlowJo version 10. For data analysis, doublets were excluded and single cells were analyzed to select lymphocytes based on their morphology by forward- versus side-scatter (FSC-SSC) dotplot. Viability was assessed by flow cytometry using LIVE/DEAD TM Fixable Violet Dead Cell Stain Kit (Thermo Fisher Scientific). For T cells, combining anti-CD8 and anti-CD4, CD8^−^CD4^+^ (CD4^+^ T cells) were identified as previously reported in our laboratory [42,44], and the percentage and MFI of Th1 (CXCR3^+^CD3^+^CD4^+^) and Th17 (CCR6^+^CD3^+^CD4^+^) wwew then analyzed (Figure S1A–C).

For macrophage subtypes, we first identified macrophages based on CD14 expression. Next, by combining anti-CD86 and anti-CD163, we were able to distinguish between M1 macrophages (high levels of CD86 and low levels of CD163) and M2 macrophages (low levels of CD86 and high levels of CD163) as previously reported [45] (Figure S1D–F). The percentage of M1 and M2 positive cells and the mean fluorescence intensity (MFI) of CD86, CXCR3, and CCR6 in each subset were obtained using FlowJo version 10 (FlowJo, Ashland, OR, USA).

### 4.4. Determination of AMPs, IL-10, TGF-β, TNF-α, and IL-17A in BAL and Culture Supernatants

BAL concentrations of lactoferrin (Assaypro LLC, St. Charles, MO, USA), SLPI (FineTest, Wuhan, China), lysozyme (Assaypro), LL-37 (HycultBiotech, Plymouth Meeting, PA, USA), IL-10 (Immunotools), TGF-β (Mabtech, Nacka Strand, Sweden), TNF-α (Mabtech), and IL-17A (PeproTech EC, London, UK) from ILD patients were determined using specific ELISA kits according to the manufacturers’ instructions and using the specific standard curves of recombinant molecules. The limits of detection were as follows: 0.313–20 ng/mL for SLPI, 62.5–4000 pg/mL for lactoferrin, 0.078–5 ng/mL for lysozyme, 0.14–100 ng/mL for LL-37, 15.62–1000 pg/mL for IL-10, 62.5–4000 pg/mL for TGF-β, 15.62–1000 pg/mL for TNF-α, and 15.62–1000 pg/mL for IL-17A.

### 4.5. Statistics

Statistical analyses were performed using GraphPad Prism 7. Normal data distribution was assessed by the Kolmogorov–Smirnov test. Variables were presented as mean ± SEM or median (interquartile range—IQR) according to normal or non-normal distribution, respectively. Comparisons between the two groups were tested with the Student’s *t*-test (paired or unpaired) or the Mann-Whitney test according to normal distribution. Comparisons of three or more groups were tested with a one-way analysis of variance (ANOVA) and the Bonferroni post hoc test. Correlation analyses were determined with Pearson’s or Spearman’s correlation according to normal distribution. ROC curves for lysozyme and SLPI levels were calculated to assign a threshold to differentiate F-ILD from NF-ILD patients. The determined area under the curve, sensitivity, and specificity were obtained. The cut-off points for lysozyme and SLPI levels were determined by an ROC curve analysis, considering the expression value that corresponded to the maximum sensitivity and specificity. *p*-values < 0.05 were considered statistically significant.

## 5. Conclusions

In conclusion, our study suggests that BAL levels of lysozyme, rather than other AMPs, could serve as a potential biomarker for diagnosing and monitoring ILD patients’ progression. Currently, we are validating the use of plasma lysozyme levels in another cohort of patients with different therapies at various time points. The final objective is to decipher whether regulating innate and adaptive immune cells by lysozyme may hold a therapeutic role in the future.

## Figures and Tables

**Table 1:** Patient characteristics. DLCO: diffusing capacity for carbon monoxide; FVC: forced vital capacity; IPF: idiopathic pulmonary fibrosis; CTD-ILD: connective tissue disease-associated interstitial lung disease: U-ILD: unclassificable interstitial lung disease; HP: hypersensitivity pneumonitis; PLCH: pulmonary langerhans cell histiocytosis; SR-ILD: smoking-related interstitial lung disease; NT: non-treated; AF: anti-fibrotic therapy; IS: immunosuppressors.

Patient Characteristics		Value
Median age (range), years		67 (38–86)
Male/female		41/22
Fibrotic status, n (%)	Fibrotic	46 (73%)
	Non-fibrotic	17 (27%)
Smoke status, n (%)	Smoker	23 (36.5%)
	Non-smoker	11 (17.4%)
	Ex-smoker	29 (46.0%)
Initial DLCO, (%)		61.1%
Initial FVC, (%)		81.2%
Etiology, (%)	IPF	19.0%
	CTD-ILD	20.6%
	U-ILD	14.3%
	HP	9.5%
	Sarcoidosis	7.9%
	PLCH	4.8%
	SR-ILD	15.9%
Treatment, (%)	NT	68.3%
	AF	6.3%
	IS	25.4%

## Data Availability

All data relevant to this study are included in the article or uploaded as Supplementary Materials. The datasets used and analyzed during the current study are included in the article. They are available from the corresponding author on reasonable request.

## Supplementary Materials

The following supporting information can be downloaded at: https://www.mdpi.com/article/10.3390/ijms25084297/s1.
