# Supplementary material for: Levels of Lysozyme and SLPI in Bronchoalveolar Lavage: Exploring Their Role in Interstitial Lung Disease
Source: Int J Mol Sci. 2024 Apr 12;25(8):4297. doi: 10.3390/ijms25084297 (PMC11050299; doi:10.3390/ijms25084297)
Supplement: Supplementary file 1 [file ijms-25-04297-s001.zip › ijms-2905081-supplementary.pdf]

Supplementary Materials

Levels of Lysozyme and SLPI in bronchoalveolar lavage: Exploring their role in interstitial lung disease

Rubén Osuna-Gómez[1], Maria Mulet[1], Silvia Barril[2-3], Elisabet Cantó[1], Paloma Millan-Billi[3-4], Ana Pardessus[3], David de la Rosa[3], Diego Castillo[3#], Silvia Vidal[1*]

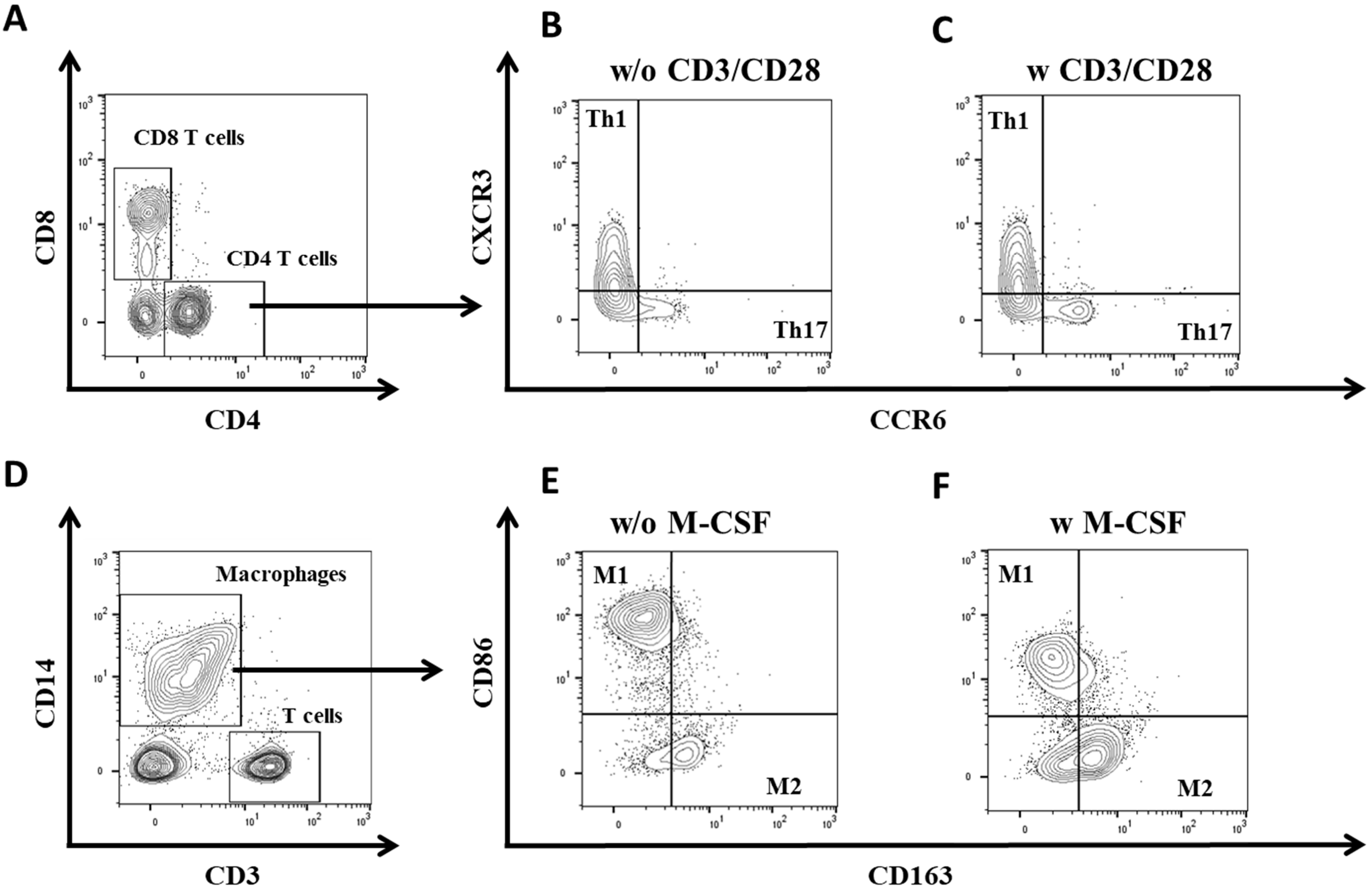

Figure S1. Gating strategies for the isolated PBMCs. (**A**) T cells were gated combining anti-CD8 and anti-CD4, CD8-$CD4^+$ ($CD4^+$ T cells) and (**B-C**) MFI of $CXCR3^{\#}$ and the MFI of $CCR6^+$ were analyzed. (**D**) Macrophages were gated by CD14 expression and (**E-F**) the percentage of $CD86^+$ and $CD163^+$ were analyzed.
